# Changes in Patient Characteristics and Early Clinical Outcomes Among Emergency Department–Admitted Inpatients During the 2024 Medical Workforce Crisis in South Korea

**DOI:** 10.3390/jcm15124804

**Published:** 2026-06-20

**Authors:** Yeon Joo Lee, Sung Woo Moon

**Affiliations:** 1 Division of Pulmonary, Allergy, and Critical Care Medicine, Department of Internal Medicine, Konkuk University Medical Center, Konkuk University School of Medicine, Seoul 05030, Republic of Korea; linnux@naver.com; 2 Division of Integrated Medicine, Department of Internal Medicine, Yonsei University College of Medicine, Seoul 03722, Republic of Korea

**Keywords:** medical crisis, emergency department, hospitalization, clinical outcomes

## Abstract

**Background/Objectives**: In February 2024, a nationwide medical crisis in South Korea caused a massive withdrawal of resident physicians. We described changes in patient characteristics and early clinical outcomes among emergency department (ED)-admitted inpatients during this disruption. **Methods**: We retrospectively analyzed 8149 internal medicine admissions via the ED at a tertiary hospital, 6 months pre- and post-crisis. Multivariable logistic regression evaluated early clinical outcomes, adjusting for baseline confounders. **Results**: Post-crisis, the internal medicine physician workforce decreased by 36%. Total admissions dropped, while patient acuity increased. After adjustment, the post-crisis group exhibited higher odds of Orders for Life-Sustaining Treatment documentation (adjusted odds ratio [aOR] 1.53, 95% confidence interval [CI] 1.34–1.74), inter-hospital transfers (aOR 1.71, 95% CI 1.49–1.96), and 48 h mortality (aOR 1.81, 95% CI 1.25–2.61). However, adjusted overall in-hospital mortality did not significantly differ (aOR 1.10, 95% CI 0.95–1.27). **Conclusions**: The crisis led to decreased admissions and higher patient acuity. Despite these shifts, adjusted in-hospital mortality did not significantly differ. This suggests that during severe workforce shortages, acute care was concentrated on a highly selected, high-acuity patient cohort, accompanied by an increased reliance on inter-hospital transfers.

## 1. Introduction

Healthcare workforce disruptions pose significant challenges to hospital systems worldwide, with the potential to affect patient access, care quality, and clinical outcomes. In February 2024, the South Korean government introduced the ‘Essential Medical Package’ to reinforce essential medical services [1]. However, medical professionals raised concerns that the policy lacked solutions to long-standing systemic issues, such as intensive workloads, disproportionate compensation, and medico-legal liability burdens [2,3]. Subsequently, South Korea experienced an unprecedented medical crisis when many disaffected doctors, including 90% of residents in all specialties, withdrew from training programs nationwide [4].

The large-scale withdrawal of health care professionals disrupted patient care, especially in tertiary hospitals, where residents are important to the frontline workforce. Before the crisis, residents, who routinely worked more than 80 h per week [2,5], formed the backbone of inpatient services in many teaching hospitals. Their sudden withdrawal forced hospitals to reorganise, recruiting physician assistants (PAs) and reducing available beds. These measures prompted concerns regarding patient safety and the resilience of the health care system [6,7], although the extent to which patient-level care processes and outcomes changed during this period has not been well characterised.

The emergency department (ED) serves as a critical interface between community-based care and inpatient hospital services and is particularly vulnerable to disruptions in hospital workforce [8]. Although the ED accounts for only 12–14% of all admissions, it is the main entry pathway for acutely ill patients [9], and disruptions in the ED-to-inpatient transition can have cascading effects on hospital operations and inpatient care. A reduced workforce can lead to extended waiting times, delayed consultations and interventions, and increased reliance on transfers to other hospitals [10,11]. In addition, crises can affect clinical decision making, influencing the frequency of admission to intensive care units (ICUs), initiation of life-sustaining treatment, and completion of ‘Orders for Life-Sustaining Treatment’ (OLSTs) [3,12,13]. Despite these concerns, empirical data describing how large-scale workforce disruptions correspond with changes in admitted patient populations and early clinical outcomes remain limited, particularly in studies including large patient samples. Understanding these clinical outcomes is critical for evaluating how hospital systems adapt acute care delivery during severe workforce shortages.

In this study, we aimed to describe changes in patient characteristics, admission patterns, and early clinical outcomes among patients admitted to the internal medicine department via the ED at a large tertiary teaching hospital in South Korea during the 2024 medical workforce crisis. This analysis offers descriptive insight into how a severe workforce disruption is associated with case-mix shifts and altered early inpatient care trajectories.

## 2. Materials and Methods

### 2.1. Study Design and Population

**Figure 1:**
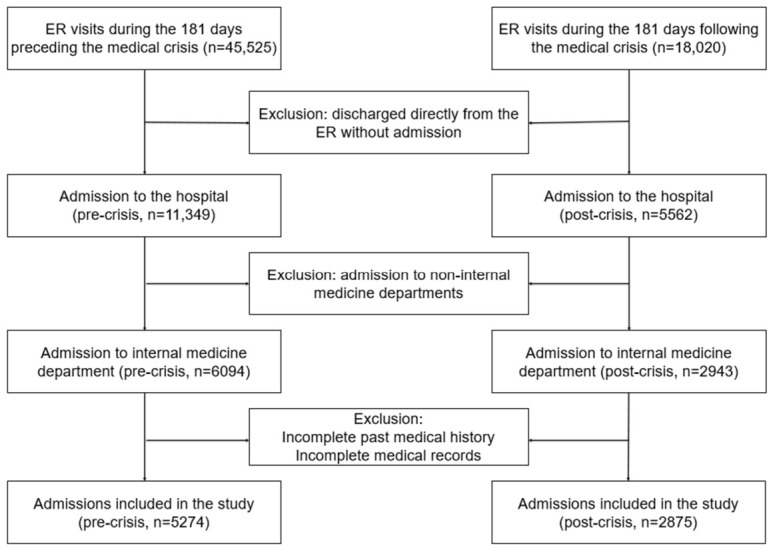
Patient selection flow diagram. Medical crisis began on 19 February 2024, when resident physicians withdrew from training programs.

On 19 February 2024, residents withdrew from the hospital, which marked the onset of a medical crisis. We identified all adult patients who visited the ED of Severance hospital during two observation periods: 181 days before the withdrawal (pre-withdrawal period) and 181 days (6 months) after the withdrawal (post-withdrawal period). Both windows were anchored to the index date (19 February 2024) and set to equal lengths to provide comparable observation time and admission volumes. This span was chosen to capture both the acute disruption and the mid-term adaptation of hospital operations and represented the maximum period for which complete data were available on both sides at study inception. A total of 45,525 ED visits were recorded before withdrawal and 18,020 after withdrawal. Patients discharged directly from the ED without admission were excluded. Among those admitted to the hospital, only those admitted to the internal medicine department were considered eligible, whereas those admitted to non-internal medicine departments were excluded. This department was selected because it represents the largest volume of emergency-to-inpatient admissions. Furthermore, as the research team is affiliated with internal medicine, this focus ensured direct and verifiable access to granular clinical data during the crisis, allowing for a more reliable analysis within a consistent clinical framework. After these exclusions, 6094 and 2943 admissions remained in the pre- and post-withdrawal periods, respectively. We then excluded admissions with incomplete records—primarily those missing the medical history needed to compute the Charlson Comorbidity Index—amounting to 820 (13.5%) pre-withdrawal and 68 (2.3%) post-withdrawal admissions. The final cohort comprised 5274 pre-withdrawal and 2875 post-withdrawal admissions (Figure 1). Because the medical crisis may have influenced patient presentation, triage, and admission decisions prior to hospitalisation, this study focused on describing differences among patients who were ultimately admitted, rather than estimating causal effects of the crisis on the general ED population.

### 2.2. Variables

The following data were extracted from the medical records: patients’ age, sex, body mass index, underlying comorbidity (measured via the Charlson Comorbidity Index [CCI]), acute-illness severity Korean Triage and Acuity Scale (KTAS) (Supplementary Table S1), primary cause of admission, time to admission decision, whether the patient required ICU care during admission, length of stay at the hospital, whether the patient was transferred to another hospital rather than completing treatment, and whether the patient was readmitted within one month (unscheduled). The CCI used in this study was calculated based on Charlson et al. [14] and the KTAS was calculated using triage records following a report developed by the Korean Ministry of Health and Welfare [15] (Supplementary Table S1). The primary diagnosis at admission was classified according to the International Classification of Diseases, 10th Revision (ICD-10), and grouped into nine categories: gastroenterology, pulmonology, cardiology, haemato-oncology, nephrology, endocrinology, infectious diseases, allergy and rheumatology, and neurology [16]. Detailed ICD-10 codes for each category are provided in Supplementary Table S2. We defined the time to admission as the interval between ED arrival and the physician’s decision to admit the patient to the internal medicine department.

### 2.3. Statistical Analysis

Continuous variables were evaluated for normality using the Kolmogorov–Smirnov test. Normally distributed variables are presented as means with standard deviations and were compared using Student’s *t*-test, while non-normally distributed variables are presented as medians with interquartile ranges (IQRs) and were compared using the Mann–Whitney U test. Categorical variables are presented as counts and percentages and were compared using the chi-squared test. To evaluate the differences in clinical outcomes between the pre- and post-crisis periods, we performed multivariable logistic regression analyses to adjust for case-mix differences. To avoid structural collider bias, the adjusted model incorporated only true pre-existing baseline confounders: age, sex, CCI, and primary diagnosis category. Variables potentially influenced by the workforce crisis, such as triage acuity and admission time, were strictly excluded from the adjustment model. Adjusted odds ratios (ORs) with 95% confidence intervals (CIs) were estimated from this model. To visually examine temporal trends in admission volume and patient acuity across the observation period, monthly counts of ED-admitted inpatients and the proportion of high-acuity patients (KTAS levels I–II) were plotted over time. Significance was defined as a two-sided *p*-value < 0.05. All analyses were performed using R version 4.4.2 (R Foundation for Statistical Computing, Vienna, Austria) and SPSS (version 29.0; SPSS Inc., Chicago, IL, USA).

## 3. Results

### 3.1. Distribution of Clinical Workforce Categories

**Figure 2:**
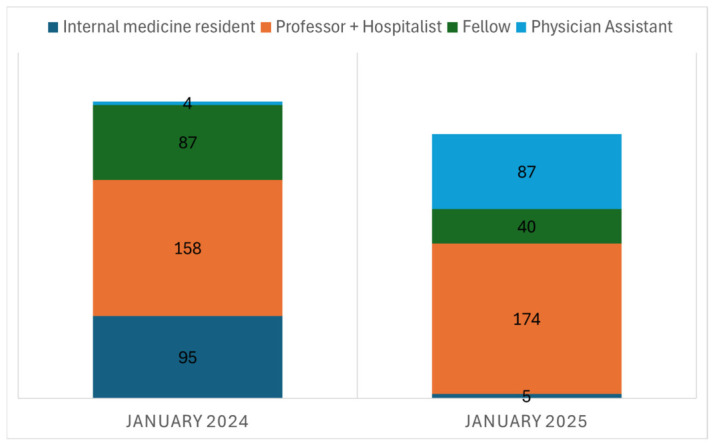
Distribution of clinical workforce in January 2024 (before medical crisis) and January 2025 (after medical crisis). Bars represent the number of providers by role, including internal medicine residents, fellows, professors/hospitalists, and physician assistants.

The composition of the clinical workforce in the internal medicine department changed substantially between the two observation periods (Figure 2). In January 2024, internal medicine residents, fellows, professors, hospitalists, and PAs provided inpatient care. Residents constituted the largest portion of the workforce, followed by fellows and attending physicians, whereas PAs accounted for a negligible proportion.

By January 2025, after the withdrawal of residents, the distribution of the workforce had changed substantially. Fewer residents and fellows were providing care, while more professors and hospitalists were filling some of the gaps left by residents. PAs, who were minimally engaged before the withdrawal, emerged as a major component of the hospital workforce in 2025, delivering a significant portion of patient care. When physicians were defined as residents, fellows, professors, or hospitalists, the total number of physicians decreased from 344 in January 2024 to 219 in January 2025, representing an overall reduction of approximately 36%.

### 3.2. Baseline Characteristics

**Figure 3:**
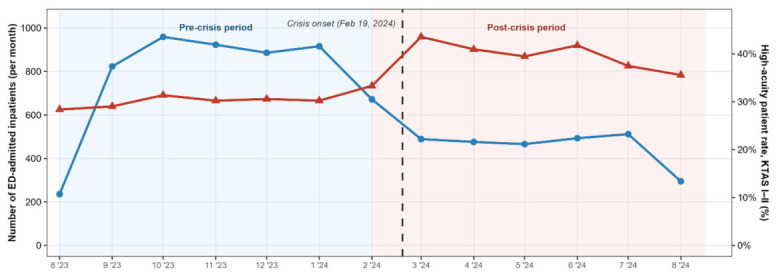
Monthly trends in ED-admitted inpatients and high-acuity patients’ rate during the 2024 medical workforce crisis. The blue line with circular markers indicates the number of ED-admitted inpatients per month (left y-axis), and the red line with triangular markers indicates the high-acuity patient rate (KTAS I–II) (right y-axis). The light blue shaded area denotes the pre-crisis period and the light red shaded area denotes the post-crisis period. ED, Emergency Department; KTAS, Korean Triage and Acuity Scale.

In total, 8149 admissions were analysed, with 5274 admissions in the pre-crisis group and 2875 in the post-crisis group. Table 1 presents the demographic and clinical characteristics of the two groups. The total number of ED–admitted hospitalisations decreased markedly during the post-crisis period, while the proportion of high-acuity patients concurrently increased. This shift occurred abruptly at the onset of the crisis in February 2024, with a concurrent marked decrease in total admission volume (Figure 3).

The reasons for hospital admission changed markedly. Post-crisis, more patients were admitted for haemato-oncology-related conditions, while hospitalisations for gastrointestinal and pulmonary diseases decreased. Consistently, the distribution of the median CCI differed significantly between groups. The process-related indicators showed marked differences. The time from arrival to admission was significantly shorter after the crisis. In contrast, the average length of hospital stay remained similar across the two periods. With respect to clinical outcomes, OLST documentation and ICU admissions were more frequent during the post-crisis period. Early mortality within 48 h of admission was significantly higher in the post-crisis group, as was overall in-hospital mortality.

### 3.3. Multivariable Analysis of Clinical Outcomes

To account for case-mix differences between periods, multivariable logistic regression analyses were performed adjusting for age, sex, and the CCI. Variables potentially influenced by the workforce crisis itself, such as triage acuity and time to admission, were excluded from the adjustment model to avoid structural collider bias. Adjusted odds ratios for clinical outcomes are presented in Table 2.

Compared with the pre-crisis group, the post-crisis group showed significantly higher rates of OLST documentation and transfers to other hospitals. Early mortality within 48 h of admission was also higher in the post-crisis group, although the absolute increase was modest. In contrast, overall in-hospital mortality did not differ significantly between periods after adjustment. Unscheduled 30-day ED revisits did not differ significantly between the two periods in the unadjusted analysis, but were significantly less frequent in the post-crisis group after multivariable adjustment.

To address potential seasonal confounding arising from asymmetric calendar coverage between the two observation periods, a sensitivity analysis was conducted restricting the cohort to February and August, the only calendar months represented in both periods (*n* = 1203) (Supplementary Table S3). The post-crisis group showed significantly higher rates of OLST documentation and transfers to other hospitals. Early 48 h mortality showed a numerically higher OR in the same direction, although statistical significance was not reached, likely reflecting reduced statistical power in the restricted sample. In-hospital mortality remained non-significant, consistent with the primary analysis. The significantly lower rate of unscheduled 30-day ED revisit observed in the primary analysis was not replicated in this restricted cohort.

## 4. Discussion

This retrospective study described changes in patient characteristics, admission patterns, and early clinical outcomes among internal medicine patients admitted via the ED during the 2024 medical workforce crisis. The post-crisis period was characterised by a marked decrease in total admissions, a higher proportion of high-acuity patients, higher comorbidity burden, and shorter times to admission decision. After multivariable adjustment, the post-crisis group showed higher odds of OLST documentation, transfers to other hospitals, and early mortality within 48 h of admission, while overall in-hospital mortality did not differ significantly between periods. The physician workforce in internal medicine decreased by 36%, and these changes were accompanied by substantial shifts in workforce composition.

Healthcare workforce disruptions are neither new [12,17] nor unique to South Korea. Nationwide workforce disruptions occurred in South Korea previously in 2000 and 2020 [12,17]; however, the present crisis is distinct, given its nationwide scope, abrupt onset, and prolonged duration. Furthermore, during the disruptions in 2000 and 2020, residents continued to provide coverage in essential areas such as the ICU and ED [17,18,19]. Strikes, walkouts, or mass resignations of healthcare providers in response to policy disputes, working conditions, or training environments have been reported in several other countries [12,13,20]. Although prior studies have reported heterogeneous effects on clinical outcomes [8,21,22,23], direct comparisons with these studies are limited by differences in healthcare systems, study populations, and contexts, and our findings should be interpreted within the specific setting of the Korean medical system and the 2024 crisis.

With the medical crisis, the hospital and remaining workforce implemented various measures to maintain patient care. Prior reports have described how remaining physicians expanded their clinical duties and worked longer hours, including additional night shifts [3,24]. Hospitals also attempted to compensate for the diminished workforce by reducing the number of available patient beds [25] and increasing the deployment of PAs. Although the early mortality rate within 48 h of admission increased in the post-crisis period, overall in-hospital mortality did not differ significantly after multivariable adjustment. However, the absence of a statistically significant difference in adjusted in-hospital mortality among admitted patients should not be interpreted as evidence that overall care delivery was fully maintained. Because the analysis is limited to patients who were ultimately admitted to the internal medicine department, the findings are conditional on admission and apply only to this specific subset.

The substantial reduction in ED visits and internal medicine admissions indicates that if admission thresholds, bed availability, or transfer decisions changed during the crisis, the post-crisis cohort represents a highly selected subset of patients. This discordance may partly reflect a shift in case mix toward patients with advanced illness who died shortly after admission, as evidenced by increased OLST documentation rates and higher acuity at presentation. Furthermore, the increased transfer rate to other hospitals after the crisis must be explicitly considered when interpreting in-hospital mortality. If patients transferred to other facilities had subsequent adverse outcomes that were not captured in our index hospital data, the observed in-hospital mortality may not fully reflect the ultimate clinical trajectories after the crisis. Therefore, the stable in-hospital mortality rate likely reflects a concentration of acute care resources on a highly selected group of admitted patients, coupled with the redistribution of critically ill patients, rather than true systemic resilience.

In this study, the number of internal medicine admissions via the ED decreased after the medical crisis. One interpretation is that access to tertiary hospital care became more constrained, with admission thresholds shifting toward higher-acuity cases. Consistent with this, the proportion of patients with higher KTAS levels and CCI scores increased after the crisis period. The concurrent increase in transfers to other hospitals may further reflect redistribution of patient care to other facilities. Beyond changes in patient presentation, it is also possible that alterations in clinical decision-making contributed to this pattern. As residents were replaced by more senior physicians, including professors and hospitalists, admission decisions may have been made more selectively, potentially raising the threshold for inpatient admission independent of patient acuity. We emphasise, however, that this remains a hypothesis: our dataset did not capture the decision-level information needed to confirm it, and it should be regarded as a direction for future study rather than an established finding.

Another important feature of the workforce withdrawal was shifting tasks to PAs, who had previously limited roles [26]. As residents withdrew, the number of PAs increased, and their practice range broadened [27]. This rapid expansion raised concerns regarding training, supervision, and the legal accountability of PAs [27]. Further studies are needed to examine the long-term impact of workforce substitution strategies.

Our findings may also inform future medical workforce planning. The reduction in the internal medicine workforce, in which residents had constituted the largest share, illustrates how heavily frontline inpatient and emergency care at tertiary teaching hospitals can depend on trainees. Although our study evaluated no workforce intervention, these observations raise the question of whether contingency measures—such as surge-staffing capacity or expanded, adequately supervised roles for non-resident providers—could mitigate the disruptions characterised here.

The lower rate of unscheduled 30-day ED revisit in the post-crisis group reached statistical significance only after multivariable adjustment, likely because the post-crisis cohort included more higher-acuity and higher-comorbidity patients who carry an inherently greater propensity for ED return visits. After accounting for this case-mix difference, the adjusted analysis revealed a modest but significant reduction in revisits, which may reflect more selective admission of patients with clearer care plans, or reduced revisit capacity due to higher transfer rates.

The two observation periods capture asymmetric seasonal patterns, with the pre-crisis period spanning late summer, autumn, and winter, and the post-crisis period covering winter, spring, and summer. To address potential seasonal confounding, we conducted a sensitivity analysis restricting the cohort to February and August, the only calendar months present in both periods (*n* = 1203), thereby eliminating seasonal confounding by design (Supplementary Table S3). The direction of effect was consistent across all outcomes, and transfer to another hospital remained statistically significant in this restricted cohort. Nevertheless, the inability to fully disentangle seasonal variation from the crisis effect using a single-year dataset remains an inherent limitation of the study design and may have also contributed to the observed differences in case acuity indicators such as the KTAS and CCI scores.

The main strength of this study is that it has shown that even within tertiary referral hospitals, which are generally considered resource-rich, measurable changes in patient admission patterns and clinical indicators can occur. Another strength is that it has used ED data, where workforce interruptions are likely immediately reflected in patient outcomes. In addition, we quantified the scale of the reduction in internal medicine workforce by providing detailed estimates.

This study has some limitations. First, the study was performed in a single tertiary referral hospital, which may limit the generalisability of our findings. Caution is warranted when generalising the results to other hospitals, centres, or countries. We initially sought collaboration with multiple centres to enhance external validity; however, potential partner institutions expressed concerns about the administrative burden and political sensitivity. Second, our study is inherently limited by selection bias. Given that the healthcare workforce crisis profoundly affected admission thresholds, hospital capacity, and transfer practices, the observed differences in outcomes may reflect substantial changes in case-mix rather than true changes in patient-level clinical trajectories. Third, while we attempted to address seasonal confounding through a sensitivity analysis restricted to overlapping calendar months, residual confounding from unmeasured indicators of acute clinical severity (e.g., physiological parameters, organ dysfunction markers) and temporal trends cannot be completely ruled out with a single-year dataset. Finally, due to changes in workforce composition and patient numbers resulting from hospital management, financial information was not available for analysis.

## 5. Conclusions

In conclusion, the 2024 medical crisis was associated with substantial shifts in workforce composition and a decrease in hospital admissions. Our findings provide descriptive evidence of observed associations between this system-level disruption and changes in early clinical trajectories, including higher patient acuity and increased hospital transfers. Although adjusted overall in-hospital mortality did not significantly differ between the periods, this finding must be interpreted cautiously. It likely reflects a concentration of acute care resources on a highly selected subset of patients and a redistribution of critically ill patients to other facilities, rather than indicating that overall care delivery was fully maintained. These results underscore the importance of considering profound case-mix shifts, altered admission thresholds, and increased inter-hospital transfers when evaluating clinical outcomes during severe healthcare workforce disruptions.

## Figures and Tables

**Table 1:** Comparison of demographic and clinical variables between pre-crisis and post-crisis groups.

	Pre-Crisis Group (*n* = 5274)	Post-Crisis Group (*n* = 2875)	*p*-Value
Age, years	65.5 ± 15.0	65.6 ± 14.4	0.592
Sex, male	2974 (56.4%)	1648 (57.3%)	0.431
Korean Triage and Acuity Scale classification			<0.001
I	236 (4.5%)	240 (8.3%)	
II	1367 (25.9%)	910 (31.7%)	
III	2536 (48.1%)	1454 (50.6%)	
IV	1031 (19.5%)	246 (8.6%)	
V	104 (2.0%)	25 (0.9%)	
Major cause of admission			<0.001
Gastroentrology	1058 (20.1%)	421 (14.6%)	
Pulmonology	878 (16.6%)	395 (13.7%)	
Cardiology	630 (11.9%)	413 (14.4%)	
Hemato-oncology	1550 (29.4%)	1095 (38.1%)	
Nephrology	398 (7.5%)	160 (5.6%)	
Endocrinology	118 (2.2%)	56 (1.9%)	
Infectious diseases	271 (5.1%)	132 (4.6%)	
Allergy & Rheumatology	51 (1.0%)	24 (0.8%)	
Others	320 (6.1%)	179 (6.2%)	
Charlson comorbidity index	2.0 (0.0–5.0)	2.0 (0.0–5.0)	<0.001
Time to admission decision, hours	9.4 (6.2–13.6)	3.7 (2.5–5.7)	<0.001
Length of hospital stay, days	8.0 (4.0–14.0)	7.0 (4.0–13.0)	0.105
OLST documentation	607 (11.5%)	501 (17.4%)	<0.001
ICU admission during admission	519 (9.8%)	468 (16.3%)	<0.001
Death within 48 h of admission	58 (1.1%)	59 (2.1%)	<0.001
In-hospital mortality	555 (10.5%)	351 (12.2%)	0.023
Death/1000 hospital days	10.4	8.6	
Unscheduled 30-day ED visit	855 (16.2%)	427 (14.9%)	0.114

OLST, Orders for Life-Sustaining Treatment; ICU, Intensive care unit; ED, Emergency department. For variables that did not follow a normal distribution (Charlson Comorbidity Index, time to admission decision, and length of hospital stay), normality was assessed using the Kolmogorov–Smirnov test, and the Mann–Whitney U test was used for between-group comparisons. These variables are presented as median (interquartile range).

**Table 2:** Odds ratios for clinical outcomes: pre-crisis versus post-crisis periods.

Outcome	Pre-Crisis (*n* = 5274)	Post-Crisis (*n* = 2875)	OR (95% CI)	*p*-Value	Adjusted OR † (95% CI)	*p*-Value
OLST documentation	607 (11.5%)	501 (17.4%)	1.62 (1.43–1.84)	<0.001	1.53 (1.34–1.74)	<0.001
Transfer to another hospital	503 (9.5%)	454 (15.8%)	1.78 (1.55–2.04)	<0.001	1.71 (1.49–1.96)	<0.001
Early mortality within 48 h	58 (1.1%)	59 (2.1%)	1.88 (1.31–2.72)	<0.001	1.81 (1.25–2.61)	0.002
In-hospital mortality	555 (10.5%)	351 (12.2%)	1.18 (1.03–1.36)	0.021	1.10 (0.95–1.27)	0.200
Unscheduled 30-day ED revisit	855 (16.2%)	427 (14.9%)	0.90 (0.79–1.02)	0.107	0.87 (0.77–0.99)	0.037

† Adjusted for age, sex, Charlson Comorbidity Index (CCI), and primary diagnosis category. OR, Odds ratio; 95% CI, 95% Confidence intervals; OLST, Orders for Life-Sustaining Treatment; ED, Emergency Department.

## Data Availability

The data presented in this study are available on request from the corresponding author. The data are not publicly available due to ethical restrictions.

## Supplementary Materials

The following supporting information can be downloaded at: https://www.mdpi.com/article/10.3390/jcm15124804/s1, Table S1: Definitions of the levels of the Korean Triage and Acuity Scale (KTAS); Table S2: ICD-10 codes used for classification of primary diagnoses; Table S3: Sensitivity analysis for seasonal confounding: clinical outcomes restricted to February and August admissions.
