# Supplementary material for: Changes in Patient Characteristics and Early Clinical Outcomes Among Emergency Department–Admitted Inpatients During the 2024 Medical Workforce Crisis in South Korea
Source: J Clin Med. 2026 Jun 20;15(12):4804. doi: 10.3390/jcm15124804 (PMC13301829; doi:10.3390/jcm15124804)
Supplement: Supplementary file 1 [file jcm-15-04804-s001.zip › jcm-4377369-supplementary.pdf]

SUPPLEMENTARY INFORMATION

Table S1. Definitions of the level of the Korean Triage and Acuity Scale (KTAS)

Level	Definition
I	Immediate aggressive treatment is needed, and life-threatening (or potentially worse) conditions.<br>Immediate medical examination must be performed.
II	Potential threats to life, limb or body function and a quick intervention is needed.<br>Physician's or nurse's revaluation must be performed within 15 min.
III	Conditions that can lead to serious problems that potentially require emergency intervention.<br>Significant discomforts or influences on physical functions in work or everyday life.<br>Physician's evaluation must be performed within 30 min.
IV	Patient's age, condition associated with the possibility of pain or worsening/complications.<br>Patient will be treated and re-verified within 1–2 h.<br>Physician's evaluation must be performed within 60 min.
V	Conditions caused by a chronic problem.<br>Sometimes there is a possibility of exacerbation, or there is a case where it is not. Some of these illnesses or injuries may be delayed or ordered to be performed.<br>Physician's evaluation must be performed within 120 min by a doctor or other hospital.

Table S2. ICD-10 codes used for classification of primary diagnoses

Category	ICD-10 codes
Gastroenterology	K00–K95
Pulmonology	J00–J99
Cardiology	I00–I99
Hemato-oncology	C00–D48; D50–D89
Nephrology	N00–N99
Endocrinology	E00–E89
Infectious diseases	A00–B99
Allergy & Rheumatology	L20–L30; M00–M99
Neurology & Others	G00–G99; F00–F99; H00–H59; H60–H95; L00–L99 (excluding allergy);<br>O00–O99; P00–P96; Q00–Q99; R00–R99; S00–T98; V01–Y98; Z00–Z99

Table S3. Sensitivity analysis for seasonal confounding: clinical outcomes restricted to February and August admissions (n=1203)

Outcome	Pre-crisis n (%)	Post-crisis n (%)	Adjusted OR† (95% CI)	p-value
OLST documentation	91 (11.9%)	74 (16.9%)	1.46 (1.04–2.06)	0.03
Transfer to another hospital	68 (8.9%)	68 (15.5%)	1.88 (1.31–2.71)	<0.001
Early mortality within 48 hours	9 (1.2%)	13 (3.0%)	2.28 (0.96–5.44)	0.062
In-hospital mortality	83 (10.8%)	54 (12.3%)	1.09 (0.75–1.59)	0.646
Unscheduled 30-day ED revisit	123 (16.1%)	58 (13.2%)	0.80 (0.57–1.12)	0.197

†Adjusted for age, sex, Charlson Comorbidity Index, and primary diagnosis category.

The Feb & Aug cohort is restricted to admissions occurring in February or August, the only calendar months represented in both observation periods.

OLST, Orders for Life-Sustaining Treatment; ED, Emergency Department.
